# Registry-Based Frequency and Clinical Characteristics of Inborn Errors of Immunity in Kazakhstan: A Retrospective Observational Cohort Study (2009–2023)

**DOI:** 10.3390/jcm14155353

**Published:** 2025-07-29

**Authors:** Nurgul Sikhayeva, Elena Kovzel, Svetlana Volodchenko, Aiganym Toleuzhanova, Gulnar Tortayeva, Gulmira Bukibayeva, Zhanar Zhussupbayeva, Marina Morenko

**Affiliations:** 1 “National Center for Biotechnology” LLP, JSC National Holding “Qazbiopharm”, Korgalzhyn 13/1, Astana 010000, Kazakhstan; 2 “University Medical Center” Corporate Fund, St. Kerey, Zhanibek Khandar Khanov 5/1, Astana 010000, Kazakhstan; elena.kovzel@umc.org.kz (E.K.); svetlanasv888@mail.ru (S.V.); aiga1999nym@gmail.com (A.T.); gulnart@bk.ru (G.T.); bukibaeva88@mail.ru (G.B.); janarali@mail.ru (Z.Z.); 3 Department of Pediatrics with Courses in Allergology, Immunology, Hematology, and Endocrinology, School of Medicine, “Medical University of Astana” NAO, Beibitshilik Street 49/A, Astana 010000, Kazakhstan; morenko_m.a@mail.ru

**Keywords:** inborn errors of immunity, congenital immune system defects, recurrent infections, autoimmune disorders, malignancies, primary immunodeficiencies, epidemiological data, Kazakhstan

## Abstract

**Background/Objectives**: Inborn errors of immunity (IEIs) represent a wide spectrum of diseases characterized by a predisposition to recurrent infections, as well as increased susceptibility to autoimmune, atopic, and autoinflammatory diseases and malignancies. The aim of this study was to report the registry-based frequency and describe the clinical characteristics of IEIs among patients in the Republic of Kazakhstan. **Methods**: We analyzed data from 269 patients belonging to 204 families who were either self-referred or referred by healthcare providers to the University Medical Center of Nazarbayev University with suspected IEIs. All patients resided in various regions across Kazakhstan. **Results**: A total of 269 diagnosed cases were identified in the national registry. The estimated prevalence was 1.3 per 100,000 population. The gender ratio was nearly equal, with 139 males and 130 females. The median age at diagnosis was 5 years (range: 1 month to 70 years), while the mean age was 11.3 years. The most common diagnosis was humoral immunodeficiency, observed in 120 individuals (44.6%), followed by complement deficiencies in 83 individuals (30.8%). Combined immunodeficiencies with syndromic features were found in 35 patients (13%), and phagocytic cell defects were identified in 12 patients (4.5%). The predominant clinical manifestations included severe recurrent infections and autoimmune cytopenias, while atopic and autoinflammatory symptoms were reported less frequently. **Conclusions**: These findings contribute to a better understanding of the registry-based distribution and clinical spectrum of IEIs in Kazakhstan and underscore the importance of early diagnosis and targeted care for affected individuals.

## 1. Introduction

Inborn errors of immunity (IEIs) are a diverse group of genetically determined diseases resulting from abnormalities in various components of the immune system [1]. These defects lead to a range of clinical manifestations, including severe and atypical recurrent infections, autoimmune disorders, and an elevated risk of developing malignancies [2]. IEIs may present as distinct syndromes or form part of broader clinical syndromes. According to the International Union of Immunological Societies, as of 2022, more than 500 congenital immune disorders have been linked to inborn errors of immunity (previously known as primary immunodeficiencies) [3]. These disorders arise from genetic mutations and can affect individuals of any age, gender, or ethnicity [4,5,6].

Despite advances in diagnostics, the true prevalence of IEIs remains underestimated, particularly in low- and middle-income countries, due to limited awareness and insufficient access to immunological and genetic testing infrastructure [7,8]. In such settings, pediatricians and general practitioners often lack familiarity with IEI manifestations and diagnostic criteria, leading to delayed or missed diagnoses [9,10]. This delay increases the risk of severe complications and long-term morbidity [11,12].

While many developed countries have established national IEI registries and integrated care networks [13], epidemiological data from Kazakhstan remain limited [14,15,16]. Although a working group was established in 2009 and a registry is currently maintained by the Inborn Errors of Immunity Center at the University Medical Center of Nazarbayev University, Kazakhstan still lacks a formal nationwide surveillance system [17].

The healthcare system in Kazakhstan faces challenges such as limited access to immunologists, underdeveloped laboratory infrastructure, and reliance on outdated diagnostic methods. These limitations contribute to underreporting and delayed recognition of IEIs, often resulting in inadequate treatment and increased healthcare burden [18,19].

This study aims to analyze registry-based data collected between 2009 and 2023, providing the first comprehensive overview of IEI cases diagnosed in Kazakhstan. By summarizing the spectrum, clinical features, and diagnostic characteristics of these patients, the study addresses a critical gap in regional IEI data and offers insights to inform national healthcare planning, resource allocation, and training initiatives.

## 2. Materials and Methods

### 2.1. Demographics and Healthcare System of Kazakhstan

Kazakhstan is a nation located in Central Asia, possessing an estimated population of around 19.9 million as of the year 2023, which encompasses a variety of ethnic groups, such as Kazakhs, Russians, Uzbeks, Ukrainians, and Uighurs, among others [19,20]. The nation employs a triadic healthcare framework—primary, secondary, and tertiary care—incorporating a national referral system to allocate patients according to the severity and complexity of their medical conditions (Supplementary File S1) [21,22,23]. The majority of patients participating in this research were directed from tertiary institutions that specialize in immunological disorders.

### 2.2. Data Collection

Patient data were obtained from the Immunology and Allergology Program of the University Medical Center at Nazarbayev University, which has systematically compiled information related to IEIs since the establishment of its national referral service. The retrospective data collection covered the period from January 2009 to December 2023 and included patients evaluated through outpatient and inpatient clinical services. Data were abstracted from institutional electronic medical records, clinical case forms, and standardized diagnostic reports used internally by immunologists during consultations. Additionally, contributions to the national patient registry were made in collaboration with the Republican Diagnostic Center (RDC) at the University Medical Center in Astana and the Scientific Center of Pediatrics and Pediatric Surgery in Almaty, which have supported the coordination and documentation of IEI cases across Kazakhstan.

Eligible cases were identified based on the program’s internal registry records, which maintain structured clinical documentation for each suspected or confirmed IEI case. These records include detailed chronological entries from initial presentation to diagnostic conclusion, ensuring traceability and consistency. Data abstraction was performed by trained personnel under supervision, using a predefined data extraction template to minimize heterogeneity and transcription errors.

Variables collected at the time of registry entry included date of referral, referral source, clinical suspicion at presentation, and diagnostic work-up performed. All demographic and clinical variables included in the analysis were pre-specified in the study protocol and de-identified at the point of extraction. Inclusion criteria consisted of (1) a confirmed IEI diagnosis primarily based on the European Society for Immunodeficiencies (ESID) or the International Consensus Document (ICON) clinical and/or immunologic criteria, where applicable; (2) completion of the diagnostic work-up; and (3) availability of sufficient clinical documentation to support case validation. In certain cases, particularly for retrospective records or regions with limited access to specialized care, national diagnostic protocols and expert clinical judgment were applied when international criteria could not be fully implemented. Patients referred from tertiary immunology centers, regional hospitals, and academic institutions were included. In addition, a small subset of patients were classified as “self-referred,” meaning they independently contacted immunologists or presented directly to the University Medical Center without formal referral, typically based on previous clinical suspicion, family history of IEIs, or unresolved immunologic symptoms. These patients underwent the same diagnostic procedures and registry review as formally referred individuals.

To ensure representativeness and avoid duplication, each patient was assigned a unique anonymized code, and only one confirmed diagnosis per individual was entered into the analytical dataset. Cases lacking complete evaluation or documentation were excluded. For subgroup analysis, patients were stratified into pediatric (≤17 years) and adult (≥18 years) cohorts based on their age at diagnosis, allowing for comparison of clinical characteristics and outcomes across age groups. Patients who underwent hematopoietic stem cell transplantation (HSCT) were included in the registry. Their data were recorded prior to transplantation, and follow-up was documented through post-transplant clinical reports when available. These patients were not excluded from the analysis unless follow-up data were entirely lacking.

### 2.3. Diagnosis and Classification of IEIs

Patients were categorized in accordance with the most recent iteration of the International Union of Immunological Societies (IUIS) classification system [3,4], which served as the principal framework for delineating IEI subtypes. Diagnostic assessments primarily followed established national protocols and, where applicable, incorporated internationally recognized clinical standards, including the diagnostic criteria delineated by the ESID and the ICON. However, in certain clinical scenarios—particularly involving retrospective records, regions with limited access to specialized immunologic diagnostics, or ambiguous cases—final classification was based on expert immunologist review in accordance with prevailing national guidelines.

In scenarios where molecular diagnostics were not accessible, the identification of humoral immunodeficiencies, including Common Variable Immunodeficiency (CVID), Specific Immunoglobulin A Deficiency (SIgAD), and agammaglobulinemia, depended on clinical history, familial background, and immunological evaluations. These evaluations comprised serum immunoglobulin concentrations (IgG, IgA, and IgM), specific antibody responses to vaccinations, and lymphocyte subset characterization via flow cytometry, particularly focusing on B-cell markers (e.g., CD19+, CD20+). Functional immunological assessments included nitroblue tetrazolium (NBT) or dihydrorhodamine (DHR) assays for neutrophil oxidative burst in chronic granulomatous disease (CGD), lymphocyte proliferation assays in response to mitogens for combined immunodeficiencies, and assessment of switched memory B cells (CD27+IgD−IgM−) using flow cytometry. Patients were analyzed by age group to account for known clinical and immunological differences in disease presentation between pediatric and adult populations.

### 2.4. Ethical Aspects

The registry was formed in accordance with the current laws of medical ethics, maintaining the confidentiality of the physician–patient relationship and the security of personal data. The data were encrypted in a computerized database while maintaining anonymity. Database management and access were reserved for physicians. In the case of genetic research, informed consent was obtained.

### 2.5. Statistical Analysis

Statistical analysis was performed at the National Center for Biotechnology (Astana, Kazakhstan). Registry-based frequencies were calculated using the population data registered at the beginning of 2023, with regional distribution determined based on patients’ home addresses. The data were analyzed using R version 4.4.0.

## 3. Results

### 3.1. Demographic Data

In 2023, the registry documented a total of 269 patients originating from 204 families throughout Kazakhstan, which correlates to a registry-derived frequency of 1.36 per 100,000 individuals within the population (Table 1). Given an approximate annual total of 395,336 live births, the minimal incidence rate of congenital disorders is projected to be 27.07 per 100,000 live births.

The age-specific distribution of cases was most pronounced among children aged 0–4 years, with 107 recorded cases (5.19 per 100,000), followed by 69 cases in the 5–9 years cohort (3.53 per 100,000) and 13 cases (0.71 per 100,000) within the 10–14 years demographic. The group consisting of individuals aged 15–17 years presented with 6 cases (0.51 per 100,000), whereas adults over the age of 18 accounted for 74 cases (0.58 per 100,000). This observed pattern may reflect improved identification and referral processes during early childhood, particularly in relation to more severe or syndromic phenotypes. In contrast, the diminished prevalence within older age brackets may be attributed to the underdiagnosis of milder or atypical cases, a lack of provider awareness in adult medicine, or historical deficiencies in referral methodologies.

**Figure 1:**
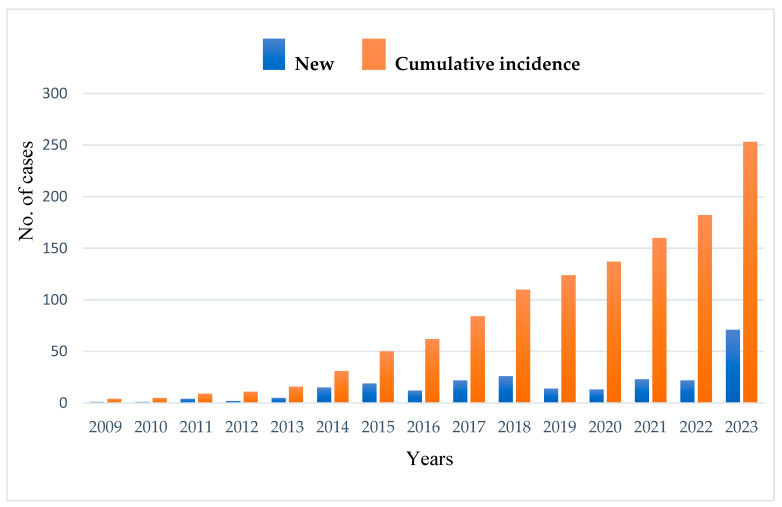
Cases of detection of IEIs in Kazakhstan from 2009 to 2023 (with new cases represented in blue and cumulative incidence in orange). From 2009 to 2013, the number of new IEI cases remained consistently low, with only a gradual increase over these years. Starting in 2014, a noticeable upward trend in new case detection began, likely reflecting improved diagnostic capabilities, increased awareness, or enhanced reporting mechanisms.

**Figure 2:**
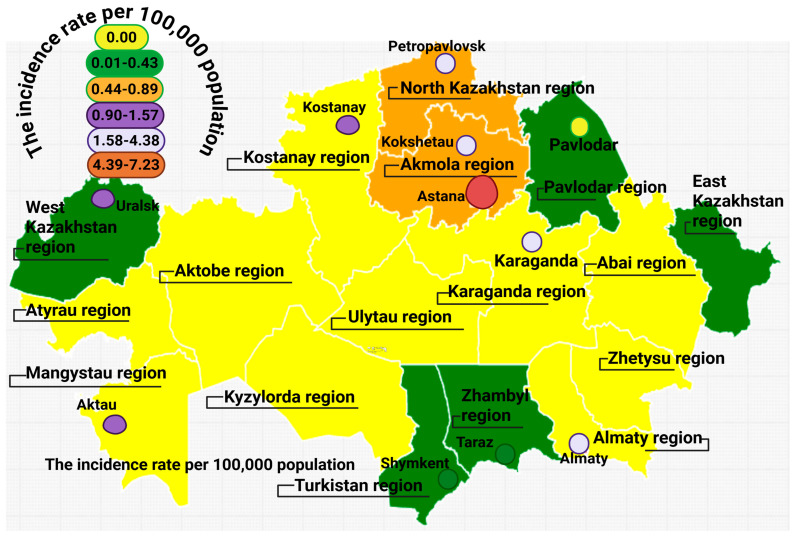
Geographical distribution of IEIs in Kazakhstan by 2023.

The average age at diagnosis was 11.3 years, ranging from 1 month to 70 years, with a median age of 5 years. Only 28% (74 cases) were diagnosed after the age of 18. The annual registry-based detection rate averaged 14 new cases, with a rising trend from 2014 to 2023 (Figure 1). The geographical distribution was uneven, with the highest number of registered cases observed in North Kazakhstan (Figure 2). This pattern might be affected by unequal access to healthcare services and differences in referral practices across regions. Patients residing near specialized facilities might experience greater access to diagnoses, while individuals in isolated or underserved regions may encounter obstacles to referral and testing. As a result, the registry-based frequency of IEIs in certain areas might be artificially low, highlighting the necessity to enhance healthcare systems, standardize referral processes, and broaden diagnostic access across the country.

The observed geographical variability in the distribution of IEI cases highlights a potential demographic bias in the registry. Patients from distant or rural areas may have limited access to healthcare services and diagnostic facilities, leading to an underrepresentation of IEI cases in these regions. This bias likely results in an overrepresentation of diagnosed cases in areas with better healthcare infrastructure, such as urban centers, while underestimating the true burden of IEIs in remote areas. To address this, it is essential to strengthen healthcare access and diagnostic capacity in rural regions, improve healthcare provider training, and consider mobile diagnostic units or outreach programs. Visual data, such as heatmaps or geographical distribution maps (Figure 2), can help illustrate these disparities and support policy recommendations aimed at improving IEI detection and management in underserved areas.

The significant rise in new IEI diagnoses noted in 2023 is primarily due to the introduction of the national hereditary angioedema (HAE) support program that began in 2022. This government-supported initiative offered standardized diagnostic protocols, featuring C1-INH functional testing and genetic screening, which greatly improved case identification nationwide. Given that HAE is categorized as a complement deficiency under the IUIS classification of IEIs, newly diagnosed HAE patients were methodically registered within the national IEI cohort. Consequently, the addition of these instances caused a significant increase in the number of newly registered IEI cases in 2023, evident from the remarkable surge observed in the registry data.

The geographical spread of IEI cases in Kazakhstan indicates a greater reported case rate in the northern and central areas, especially in the vicinity of the capital and surrounding cities. These regions house the primary tertiary care facilities that provide specialized services in immunology and genetic diagnostics. While the registry documents patients’ true living addresses, the availability of well-equipped facilities enhances the chances of diagnosis and entry into the registry. In contrast, the decreased recorded incidence observed in the southern and western areas is likely due to inadequate healthcare facilities, a lack of trained professionals, and diminished public awareness. Although rates are expressed per 100,000 individuals, the patterns seen (Figure 2) reflect diagnostic inconsistencies across different regions and underscore the need to enhance healthcare access and outreach initiatives in underserved areas.

**Figure 3:**
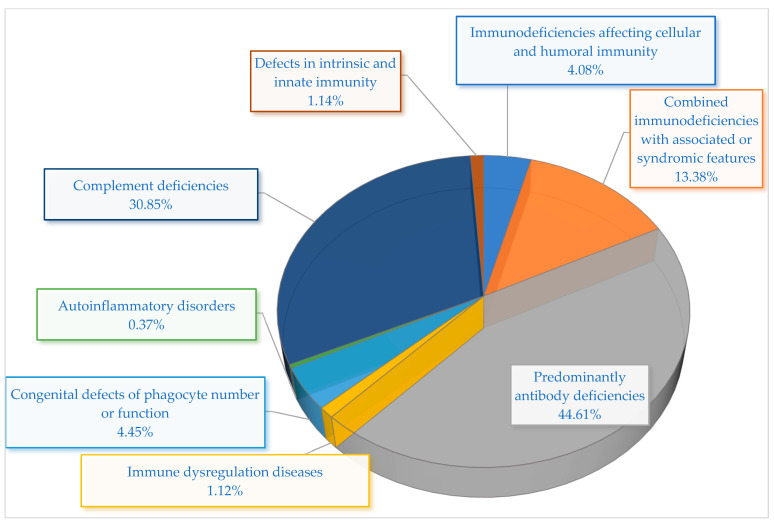
Distribution of PID categories among 269 patients diagnosed in Kazakhstan.

The prevalence of IEIs in Kazakhstan shows significant variation among immunological categories and disease subtypes (Table 2, Figure 3). The most common category was mainly predominantly antibody deficiencies (PADs), making up 44.72% of all instances. Among this cohort, the most prevalent diagnosis was common variable immunodeficiency (CVID), accounting for 16.73% of patients, with selective IgA deficiency (SIgAD) closely following at 15.99%. Furthermore, functional B-cell deficiency represented 8.18%, whereas Bruton’s agammaglobulinemia was found in 3.72% of instances. These statistics emphasize the impact of humoral deficiencies in the Kazakhstani IEI group and align with worldwide patterns in IEI registries.

Among adult patients, the most frequently detected categories were complement deficiencies and PADs. Complement deficiencies represented the second most prevalent category overall, accounting for 30.86% of all recorded cases, with all instances resulting from C1 inhibitor (C1-INH) deficiency. This elevated frequency is probably connected to the recent nationwide emphasis on enhancing the diagnosis and treatment of HAE.

Syndromic manifestations linked to combined immunodeficiencies made up 13.38% of total cases. Hyper-IgE syndrome (HIES) was the most prevalent (4.09%), followed by ataxia–telangiectasia (A-T) (3.35%), DiGeorge syndrome (DGS) (2.97%), Wiskott–Aldrich syndrome (WAS) (2.60%), and Nijmegen breakage syndrome (NBS) (0.37%). These syndromic types are frequently identified in pediatric patients because of their unique clinical phenotypes and related systemic manifestations.

Immunodeficiencies impacting both cellular and humoral immunity were identified in 4.09% of individuals. This group was primarily affected by severe combined immunodeficiency (SCID), which represented 3.72%, along with one instance of major histocompatibility complex (MHC) Class II deficiency. Significantly, SCID exhibited the highest linked mortality rate (50%) among all categories.

Other IEI categories were clinically significant but less frequent. Congenital abnormalities in the number or function of phagocytes were responsible for 4.46% of cases, all identified as chronic granulomatous disease (CGD). Diseases of immune dysregulation, such as autoimmune lymphoproliferative syndrome (ALPS) and autoimmune polyendocrinopathy–candidiasis–ectodermal dystrophy (APECED), accounted for a total of 1.11% of cases. Deficiencies in intrinsic and innate immunity accounted for another 1.11%, comprising individuals with Toll-like receptor 3 (TLR-3) deficiency and chronic mucocutaneous candidiasis (CMC). Finally, one instance of familial cold autoinflammatory syndrome (FCAS) was documented, representing 0.37% of the group.

A sum of 195 pediatric cases was documented, indicating considerable differences in disease occurrence among IEI categories (Table 3). The most common category was complement deficiencies, representing 25.13% of pediatric IEI instances. All 49 instances were identified as having C1 inhibitor deficiency, showing a male-to-female ratio of 6:43 and a 2% mortality rate (1/49), with death attributed to laryngeal edema before the initiation of appropriate prophylactic treatment. This discovery highlights the significance of early detection of HAE in children.

Mainly, antibody deficiencies were the second most common category, accounting for 44.86% of cases. In this group, CVID and SIgAD each represented 12.31%, while functional B-cell deficiency and Bruton’s agammaglobulinemia contributed 11.28% and 5.13%, respectively. Among CVID cases (*n* = 24), one death was recorded in an untreated patient with chronic lung disease, reflecting a 4% mortality rate. SIgAD cases were primarily identified during immunologic evaluation for recurrent infections, allergic conditions, or autoimmune features. In addition, a smaller subset was diagnosed incidentally during broader immunologic screening for suspected IEIs. Due to the retrospective nature of the registry, we were not always able to distinguish between symptomatic and incidental diagnoses. No associated deaths were recorded for SIgAD or functional B-cell deficiency. Of the 10 patients with Bruton’s agammaglobulinemia, two deaths were documented (20%), and both patients lacked access to regular immunoglobulin therapy.

Combined immunodeficiencies with syndromic features accounted for 18.58% of pediatric cases. The most prevalent of these included hyper-IgE syndrome (5.64%), DiGeorge syndrome (4.10%), ataxia–telangiectasia (3.34%), and WAS (3.59%). Among patients with ataxia–telangiectasia (*n* = 6), a mortality rate of 22% (1/6) was observed due to progressive pulmonary failure. However, the average age of this subgroup at the time of data collection was 10.7 years, and longer-term follow-up is needed to accurately assess disease-related survival. In DiGeorge syndrome (*n* = 8), two deaths (25%) were recorded—both related to cardiac anomalies and severe infections in infancy. These findings reflect the multisystem involvement and progressive characteristics of these conditions.

Immunodeficiencies that impacted both cellular and humoral immunity were noted in 5.63% of instances, comprising SCID (5.12%) and a single case of MHC Class II deficiency (0.51%). SCID remained the deadliest IEI subtype, with a mortality rate of 50% (5/10). All deceased patients either lacked access to HSCT or experienced treatment delays.

Alternative, rarer categories comprised congenital abnormalities in phagocyte count or function (6.15%), all identified as CGD, with a mortality rate of 8.3% (1/12) due to sepsis in an untreated infant; immune dysregulation disorders (1.54%) like ALPS and APECED, with both exhibiting 0% mortality; and deficiencies in intrinsic and innate immunity (1.54%), which included TLR-3 deficiency and chronic mucocutaneous candidiasis. One instance (0.51%) of FCAS was noted in the pediatric category, with no associated complications or mortality.

The category most commonly identified in adults was complement deficiencies, representing 45.95% of all adult IEI instances (Table 4). Every one of the 34 patients in this cohort was identified as having C1 inhibitor deficiency, highlighting the ongoing clinical impact of HAE in adults. Of these, 25 cases were confirmed through genetic testing, revealing a modest mortality rate of 3%, emphasizing the significance of ongoing disease monitoring and management. The majority of adult IEI cases were predominantly antibody deficiencies, accounting for 54.05%. In this category, CVID was the predominant subtype, accounting for 28.38% of cases. It showed an almost identical male-to-female ratio (11:10), with 14 out of 21 cases genetically verified and no deaths reported. At the same time, SIgAD represented 25.68% of adult instances, showing a significant female majority (4:15). Importantly, none of the SIgAD cases received genetic confirmation, and there were no fatalities linked to this condition.

In total, 169 of 269 patients (62.8%) underwent genetic testing. Selection criteria for genetic testing included early-onset disease, syndromic presentation, consanguinity, or poor response to standard treatment. Genetic examination of patients from Kazakhstan with IEIs showed significant diversity in the genes involved, types of variants, and inheritance patterns across various IEI subtypes (Table 5). The majority of genetic tests were conducted in patients with clinically severe phenotypes or syndromic features. In SCID patients, variants in genes like *IL2RG*, *JAK3*, and *RAG1* were identified, with the predominant one being c.678delA, an X-linked pathogenic mutation indicative of hemizygous expression. A case of MHC Class II deficiency was linked to a heterozygous *CIITA* variant (c.1992+1G>A), indicating autosomal recessive inheritance. Combined immunodeficiencies alongside syndromic characteristics were associated with pathogenic mutations in *DOCK8*, *STAT3*, *WAS*, *ATM*, and *TBX1*, which included an autosomal dominant *STAT3* variant (c.1144C>T; p.Arg382Trp) and an X-linked *WAS* variant (c.271C>T). Individuals with ataxia–telangiectasia possessed a homozygous *ATM* variant, validating autosomal recessive inheritance. In primarily antibody deficiencies, *BTK* mutations (e.g., c.1483G>A) were frequently identified in Bruton’s agammaglobulinemia, whereas CVID instances displayed various variants like c.310T>C (p.Cys104Arg) in *TNFRSF13B*, commonly regarded as variants of uncertain significance. No causative variant was found for SIgAD, highlighting persistent difficulties in genetic diagnosis. Immune dysregulation syndromes such as APECED and ALPS were linked to pathogenic variants of *AIRE* and *FAS*, both of which are inherited in a dominant manner. Autosomal dominant mutations in BLNK were associated with functional B-cell deficiency. Patients with CGD exhibited X-linked mutations in *CYBB*, whereas cases of FCAS and HAE were linked to autosomal dominant mutations in *NLRP3* and *SERPING1*, respectively. Moreover, defects in intrinsic and innate immunity, including TLR-3 deficiency and chronic mucocutaneous candidiasis, were linked to pathogenic variants in *TLR3* and *STAT1*, respectively, through autosomal inheritance.

### 3.2. Geographical Distribution

The evaluation of the geographical distribution of IEIs in Kazakhstan relies on a group of patients from the national IEI registry who had thorough details regarding their diagnosis and residence at the time of the analysis. From the overall group of 269 patients, 204 met these criteria and were involved in the regional case rate assessments. Currently, diagnosed case rates per 100,000 population are available for 15 cities throughout Kazakhstan (Figure 2). The highest rate of documented cases was observed in Astana, with figures ranging from 4.39 to 7.23 per 100,000 residents. Intermediate registry-derived detection rates, which varied from 1.58 to 4.38 per 100,000, were recorded in six cities, including Almaty and various cities in the Akmola region (like Kokshetau, Petropavlovsk, and Stepnogorsk), along with Pavlodar and Karaganda. In the western areas, towns such as Aktobe, Uralsk, and Aktau had diagnosed case rates ranging from 0.90 to 1.57 per 100,000. In the Abai region, Semey showed a rate between 0.44 and 0.89 per 100,000, whereas the lowest figures, between 0.01 and 0.43 per 100,000, were noted in Taraz, Shymkent, and Ust-Kamenogorsk. It is important to highlight that regional case rate evaluations are constrained by incomplete reporting and missing residential information in some of the registry records. Moreover, the number of diagnosed cases may be underestimated in remote areas because of limited access to specialized immunological care and diagnostics. Higher detection rates were observed in areas situated near major immunological centers, highlighting the role of healthcare infrastructure and referral systems in influencing case documentation. Despite efforts to ensure geographic representativeness, continuous improvements in case reporting and regional outreach remain essential for strengthening future epidemiological assessments.

### 3.3. Clinical Characteristics

The registry-documented rate among males was slightly higher (1.44 per 100,000) compared to females (1.28 per 100,000), as shown in Table 1. In addition to these documented case patterns, the average age at diagnosis for patients with IEIs varied according to the IUIS classification: 3.1 years for combined IEIs with syndromic manifestations, 8.7 years for antibody deficiencies, 5.0 years for phagocytic system defects, 1.9 years for SCID, 28.2 years for complement deficiencies, 2.3 years for innate immunity defects, 4.5 years for immune dysregulation diseases, 3.0 years for autoinflammatory disorders, and 2.5 years for phenocopies of IEIs. The median age of 1.9 years for SCID is unexpectedly high, likely reflecting diagnostic delays associated with limited newborn screening and insufficient awareness among healthcare providers in Kazakhstan.

Recurrent infections were the predominant clinical feature, affecting 66% of patients. Among these, 47% experienced recurrent lower respiratory tract infections, 18% had upper respiratory tract infections, and 9% had suppurative or fungal skin infections. Additionally, 10% were diagnosed asymptomatically through family history and screening. Non-infectious complications were also common: growth retardation and developmental delays were observed in 47% of patients, autoimmune manifestations in 16%, severe chronic relapsing atopic dermatitis in 6.3%, and recurrent non-infectious angioedema in 30.8%.

Severe infections included recurrent bacterial pneumonia, chronic otitis media, and persistent fungal skin infections. One case involved a child with SCID who experienced recurrent fungal infections of the oral mucosa and skin. Another case involved a patient with CGD who suffered multiple abscesses due to *Staphylococcus aureus*.

Hospitalizations occurred in 13.3% of patients due to severe infections such as sepsis, osteomyelitis, or meningitis. Malignancies, particularly lymphomas and leukemias, were the second most common cause of death after infections. In HAE, asphyxiation from mucosal swelling was a frequent cause of mortality.

Vaccination-related complications were identified in 25 patients. The most common events were disseminated *Bacillus Calmette–Guérin* (BCG) infections and vaccine-associated paralytic poliomyelitis (Table 6).

## 4. Discussion

In 2023, Kazakhstan reported a registry-derived frequency of IEIs of 1.36 per 100,000 individuals and a minimal incidence of 27.07 per 100,000 live births. While this reflects some improvement in national detection, these values remain modest compared to international data [24,25,26,27,28,29,30,31,32]. For example, Germany reports a minimum prevalence of 2.72 per 100,000 [31], and Ireland shows an adult IEI prevalence of 8.85 per 100,000 [32]. However, the reported case rate of IEIs in Kazakhstan is lower than that observed in developed nations. For instance, Kazakhstan’s rate is considerably lower compared to Norway (5.03 per 100,000), France (4.4 per 100,000), the United States (5.01 per 100,000), Australia (4.67 per 100,000), and Canada (9.78 per 100,000). These differences may be influenced not only by ethnic and geographic factors, as highlighted in various epidemiological studies, but also by variations in healthcare systems [33,34,35,36,37,38]. In the Russian Federation, the prevalence is similar, at 1.3 per 100,000, though the estimated birth incidence is lower, at 5.7 per 100,000 live births [27]. In Latin America, a multicenter study documented 206 confirmed patients, with a prevalence of 0.97 per 100,000 [24,25,28], while a broader regional registry included 9307 patients, mostly from Argentina, Brazil, Mexico, and Colombia [26]. In the Middle East and North Africa (MENA) region, IEI prevalence varied considerably across countries by 2021. High rates were reported in Turkey (7.58 per 100,000; 6392 patients; population 84.3 million), Kuwait (7.75; 331 patients; population 4.27 million), Iran (6.41; 5384 patients; population 83.9 million), Tunisia (6.03; 710 patients; population 11.27 million), Jordan (5.46; 544 patients; population 9.95 million), and Qatar (4.92; 137 patients; population 2.78 million). Moderate prevalence was observed in Oman (3.83) and Sudan (3.32), and the MENA regional average was 2.96. Lower values were recorded in Algeria (2.36), Azerbaijan (1.38), Morocco (1.87), Libya (1.65), Saudi Arabia (1.59), and Lebanon (0.83). Extremely low rates were seen in Bahrain (0.17), Syria (0.09), Iraq (0.02), Afghanistan (0.02), and Yemen (0.01), likely due to underdiagnosis or incomplete registry coverage [29]. Kazakhstan’s relatively low prevalence must be interpreted cautiously. The registry system is not yet fully developed, access to diagnostic services remains limited, and the country’s population of around 20 million is significantly smaller than that of most of the countries referenced. Moreover, disparities between urban and rural areas contribute to underreporting and diagnostic delays. Although detection has improved in recent years, particularly in cities with access to specialized care, nationwide diagnostic coverage and reporting infrastructure still require substantial development [30].

The gender distribution within our cohort exhibited a nearly equitable ratio, comprising 139 males and 130 females. The analysis of age distribution indicated a pronounced prevalence of diagnoses during early childhood. Among the total of 269 patients, the highest occurrence was documented in the demographic aged 0–4 years, with 107 instances (5.19 per 100,000), followed by 69 instances (3.53 per 100,000) within the 5–9-year age bracket. Diagnoses exhibited a significant decline in older children and adolescents, with 13 cases (0.71 per 100,000) recorded in the 10–14-year age group, and a mere 6 cases (0.51 per 100,000) noted in the 15–17-year cohort. Adults (≥18 years) represented 74 cases (0.58 per 100,000), accounting for 28% of the overall population studied. These patterns imply a potential underdiagnosis within older demographics, possibly attributable to attenuated clinical manifestations or a deficiency in specialized care within adult healthcare settings. In comparison to international statistics, our results align with trends observed in the Russian Federation, where 88% of immunodeficiency and immune dysregulation cases were diagnosed prior to reaching 18 years of age, with only 12% identified in adult patients [28,39]. In the MENA region, the median age at which symptoms first manifested was documented at 36 months, with only 3.2% of individuals presenting after the age of 15 [27,29]. Likewise, data from the Latin American registry revealed that 78.16% of participants were 18 years or younger at the time of inclusion, with over 55% being under 10 years of age [28]. These comparisons indicate that the detection of pediatric cases in Kazakhstan is nearing regional benchmarks; however, the identification of adult cases remains markedly constrained. The comparatively elevated average age at diagnosis within our cohort (mean 11.3 years; median 5 years) suggests that numerous children are diagnosed at a delayed stage, particularly within rural locales that have constrained access to specialized medical care. Enhancing early referral mechanisms and augmenting awareness of adult immunology are essential priorities.

The geographic spread of IEI cases in Kazakhstan shows significant regional variations, with the highest incidence rates found in Astana (4.39 to 7.23 per 100,000 inhabitants) and moderate rates in Almaty, Kokshetau, Petropavlovsk, and Karaganda (1.58 to 4.38 per 100,000). In contrast, regions in the south and east such as Taraz, Shymkent, and Ust-Kamenogorsk displayed significantly lower detection rates, ranging from 0.01 to 0.43 per 100,000. These differences reflect disparities in healthcare infrastructure, limited access to trained immunologists outside urban centers, and inconsistent referral mechanisms. In rural and remote areas, patients often remain undiagnosed due to a lack of specialized care and insufficient awareness among healthcare providers. Although advanced diagnostic tools such as flow cytometry, targeted gene panels (TGPs), whole-exome sequencing (WES), and clinical exome sequencing (CES) are available, their use is primarily confined to tertiary-level immunology laboratories located in Astana and Almaty. Basic immunophenotyping is included in the national healthcare guarantee program; however, access to comprehensive molecular diagnostics remains limited outside major cities. Similar regional disparities have been reported in the Russian Federation, where most cases are diagnosed in urban centers and some federal districts report few or no registrations despite having comparable populations [26]. In Sri Lanka, nearly all confirmed IEI cases were diagnosed at the national referral center in Colombo, with very few identified in rural districts, highlighting the impact of centralized care [27]. To overcome these challenges, Kazakhstan must expand immunology services beyond urban areas, enhance diagnostic capacity in regional hospitals, and implement mobile diagnostic teams. Successful models from other countries suggest that geospatial heatmaps and standardized referral protocols can help ensure equitable access to diagnosis and treatment for patients with IEIs.

In our cohort, the largest immunological category was antibody deficiencies (44.72%), consistent with global findings where humoral defects are the most commonly reported IEI subtype, especially among adults [39]. CVID (16.73%) and SIgAD (15.99%) were prevalent in this group, indicating diagnostic trends similar to those in European registries. Complement deficiencies constituted the second most prevalent category (30.86%), all linked to C1 inhibitor deficiency, demonstrating a strong national initiative in HAE diagnosis, akin to recent registry patterns observed in nations like Norway and Italy, where the reporting of complement defects has risen owing to improved HAE awareness and accessibility to genetic tests [28,40,41,42,43]. Significantly, combined immunodeficiencies with syndromic characteristics represented 13.38%, emphasizing the importance of diagnostics in pediatric patients exhibiting clear clinical phenotypes. Although SCID represented 3.72% of the entire cohort, it had a mortality rate of 50%, highlighting the critical necessity for newborn screening and prompt therapeutic intervention.

A comparative study of disease-specific prevalence among national registries reveals significant differences in the immunological range of IEI, primarily influenced by diagnostic ability, registry development, and genetic patterns unique to populations. In Kazakhstan, the most common category of immunodeficiency disorders was antibody deficiencies, representing 44.72% of cases, which included CVID (16.73%), SIgAD (15.99%), functional B-cell deficiency (8.18%), and Bruton’s agammaglobulinemia (3.72%) as the main types. These results align with information from Germany, where CVID made up 30% of 1825 PID patients, followed by unclassified antibody deficiencies at 11% and agammaglobulinemia at 5% [32]. Likewise, in the Russian registry, antibody deficiencies accounted for 26% of all instances, reinforcing the global prevalence of humoral deficiencies in IEI groups [27,44]. Complement deficiencies were significantly heightened in Kazakhstan, making up 30.86% of all IEIs, mainly as a result of C1 inhibitor deficiency (HAE). This corresponds with Iran (*n* = 114), Saudi Arabia (29), Tunisia (34), and Kuwait (23), where increased prevalence probably indicates ongoing national HAE diagnostic efforts [29,45,46,47,48,49]. Data from Kazakhstan’s pediatric population indicated a 25.13% rate of complement deficiencies, highlighting the effects of early onset and supporting the need for broader pediatric screening initiatives. In Kazakh cases, combined immunodeficiencies and syndromic forms accounted for 13.38%, which included hyper-IgE syndrome (4.09%), ataxia–telangiectasia (3.35%), DiGeorge syndrome (2.97%), and WAS (2.60%). Similar rates were observed in MENA countries like Iran (CID: 684; syndromic CID: 926), Turkey (CID: 375; syndromic CID: 471), and Algeria (CID: 363; syndromic CID: 197), reflecting an equivalent diagnostic acknowledgment of systemic and syndromic IEIs [29].

Uncommon IEI categories in Kazakhstan, such as phagocytic defects (4.46%), immune dysregulation (1.11%), defects in innate immunity (1.11%), and autoinflammatory syndromes (0.37%), continued to be diagnosed infrequently, probably due to limitations in genetic diagnostics. Conversely, MENA nations such as Turkey and Iran documented markedly elevated instances of immune dysregulation (650 and 119 cases, respectively) and autoinflammatory disorders (71 and 1013, respectively), indicating improved molecular diagnostics and registry comprehensiveness [29,50]. Differences in the age of diagnosis further highlight systemic challenges. In Kazakhstan, SCID was identified at a median age of 1.9 years, which is late for a condition that necessitates early neonatal detection. Complement deficiencies were identified at an average age of 28.2 years, indicating patterns of onset in adulthood. Antibody deficiencies (average 8.7 years) and syndromic combined immunodeficiencies (3.1 years) also showed delays in diagnosis. This trend aligns with results from the Russian registry, which indicated that the median diagnostic delay varied between 4 months and 11 years based on the subtype, showing greater delays for adult-onset IEIs such as CVID and HAE [27,51].

The greatest levels of genetic confirmation were noted in syndromic immunodeficiencies and innate immune deficiencies, aligning with previous research from ESID and Iranian IEI registries, which prioritized these subtypes for sequencing based on severe clinical cases or possible familial transmission [28,29]. The use of specific gene panels in Kazakhstan, together with the national HAE diagnostic initiative, has facilitated the detection of actionable variants and improved diagnostic accuracy. Nonetheless, there are still gaps in the molecular diagnosis of humoral deficiencies like CVID and SIgAD, where variants were identified less often, highlighting global issues linked to polygenic causes or unidentified causative genes [30].

Mortality patterns in IEI patients in Kazakhstan generally mirror global trends but also underscore key disparities when compared to international registry data. In Kazakhstan, the highest disease-specific mortality was observed in patients with SCID (50%), followed by DiGeorge syndrome (25%), ataxia–telangiectasia (22%), and Bruton’s agammaglobulinemia (20%). These outcomes are largely attributed to late diagnosis and limited access to HSCT or immunoglobulin replacement therapy. The mortality burden associated with SCID and mixed cellular–humoral IEIs, which together accounted for 4.09% of all IEIs (with SCID alone comprising 3.72%), highlights a pressing need for early diagnosis. This finding aligns with data from the LASID, which reported mortality rates of 40.82% for T-B- SCID subtypes and 29.27% for T-B+ SCID variants, with Omenn syndrome reaching 52.6% [28,49]. Similarly, LASID data indicated mortality rates of 13.91% and 13.09% for ataxia–telangiectasia and WAS, respectively [28,49], reinforcing the severity of syndromic IEIs, which accounted for 13.38% of Kazakhstani cases. The MENA registry also reported high mortality in non-syndromic combined immunodeficiencies (51.7%) and syndromic CID (22.5%), particularly in patients with *RFXANK*, *RAG1*, and *IL2RG* mutations, where the most common causes of death were infection and respiratory failure [29]. These circumstances are echoed in Kazakhstan, where pneumonia, sepsis, and BCG-related infections were prevalent among fatal cases. In contrast, Russia’s national registry indicated an overall mortality rate of 9.8% across 2051 patients, with 63% of IEI-related deaths occurring before the age of five—primarily due to SCID, syndromic CID, phagocytic defects, and immune dysregulation [27]. Meanwhile, the German National PID registry reported a significantly lower overall mortality of 2%, with deaths typically resulting from infections (39%), respiratory failure (31%), and organ failure (25%), although SCID and CGD remained leading causes of early mortality even in the context of HSCT availability [32]. Taken together, the mortality profile of IEIs in Kazakhstan resembles that of resource-constrained regions such as MENA and Latin America more closely than that of Western Europe, underlining the urgent need for national neonatal screening programs and equitable access to curative therapies.

Across all registries, delays in diagnosis and access to advanced care were consistently emphasized and are particularly evident in Kazakhstan’s cohort. In Kazakhstan, the median age for diagnosing SCID was 1.9 years—considered late for a condition that usually appears in infancy—whereas the diagnostic delay for complement deficiencies extended to 28.2 years. Comparable gaps were noted in MENA nations, with a median delay of 41 months across IEIs, particularly in countries exhibiting poor DALY indicators and lacking national registry systems [29]. In Russia, wait times varied from 4 months to more than 11 years based on the illness, with the longest diagnostic delays noted in patients with A-T and PAD [27]. Conversely, Germany’s enhanced monitoring and diagnostic frameworks facilitated the prompt recognition of IEI patients, such as those with CVID and CGD, although late fatalities still happened (up to age 88 years) [32]. International comparisons provide additional context for Kazakhstan’s status: from 2013 to 2020, the number of diagnosed IEI patients in MENA rose by 19.6%, while Australia recorded a 1961% increase, Latin America 64%, and the USA 32% [29]. Additionally, just 3.2% of patients in MENA received a diagnosis in adulthood, significantly lower than the rates noted in ESID (23.8%), USIDNET (32%), and JMF (36.1%) [29]. Kazakhstan probably experiences this lack of representation in adult diagnoses, particularly for CVID and HAE. This emphasizes the necessity for comprehensive newborn screening across the country, expanded genetic testing, and organized follow-up for adult immunology. In the absence of these reforms, mortality from IEIs in Kazakhstan will probably continue to match that of resource-constrained countries, despite new diagnostic initiatives.

The genetic diversity seen in IEI patients in Kazakhstan shows considerable heterogeneity, with pathogenic variants found in various immunological classifications. In SCID patients, mutations in *IL2RG*, *JAK3*, and *RAG1* were most commonly identified, notably a recurrent X-linked mutation in *IL2RG* (c.678delA), aligning with global registries that indicate *IL2RG* as one of the frequently involved genes in X-linked SCID [5,18,29]. A case of MHC Class II deficiency was linked to a *CIITA* splicing variant (c.1992+1G>A), supporting earlier findings of autosomal recessive inheritance patterns in this uncommon yet serious type of IEI [5,32,39]. Syndromic combined immunodeficiencies are characterized by mutations in genes such as *STAT3*, *DOCK8*, *ATM*, *WAS*, and *TBX1*, including the dominant-negative *STAT3* variant (c.1144C>T, p.Arg382Trp) and the X-linked *WAS* mutation (c.271C>T), both recognized as established causes of hyper-IgE syndrome and WAS, respectively [5,29,39]. Bruton’s agammaglobulinemia was associated with classic *BTK* mutations like c.1483G>A, whereas functional B-cell deficiency was connected to *BLNK*, indicating issues in early B-cell development [27]. Despite the identification of *TNFRSF13B* variants (such as c.310T>C, p.Cys104Arg) in individuals with CVID, these variants were primarily categorized as VUS, reflecting the persistent diagnostic challenges in this subtype. Among the 45 genetically tested CVID patients (75.6% of the total CVID cohort), only one individual harbored a confirmed pathogenic variant (in *PIK3R1*), while the remaining cases predominantly involved VUS in genes such as *TNFRSF13B*, *CR2*, and *PI3KCG*. Given that pathogenic variants in PIK3R1 are associated with Activated PI3K Delta Syndrome type 2 (APDS2), this case may potentially represent a misclassified APDS rather than CVID. However, due to insufficient clinical and immunophenotypic data at the time of registry entry, the diagnosis remained as CVID, with future re-evaluation warranted. This highlights the complexity of CVID genetics, where many heterozygous variants—some in rarely implicated genes—lack sufficient clinical or functional evidence to establish a definitive diagnosis. Significantly, no pathogenic variants were found in SIgAD patients, consistent with studies indicating the probable polygenic or multifactorial aspects of SIgAD pathogenesis. Genetic validation in immune dysregulation syndromes was accomplished by detecting autosomal dominant mutations in *AIRE* and *FAS*, whereas innate immune deficiencies and autoinflammatory conditions were associated with *TLR3*, *STAT1*, *NLRP3*, and *SERPING1*, aligning with findings from other local and global groups [28,29,52]. The variation in gene mutations and inheritance patterns in Kazakhstani patients emphasizes the necessity of incorporating NGS methods into standard diagnostic procedures, particularly for complex or unusual IEI phenotypes.

Nevertheless, complement deficiencies accounted for 30.8% of our cohort, which is markedly higher than the 2% reported in the ESID registry [31]. This discrepancy is likely attributable to the recent implementation of a government-sponsored national diagnostic and support program for HAE in Kazakhstan, launched in 2022. As part of this initiative, all patients with suspected HAE undergo comprehensive diagnostic evaluation, including assessment of both the quantitative and functional levels of C1 inhibitor, measurement of C3 levels, and genetic testing for *SERPING1* mutations. Upon diagnostic confirmation, patients are provided with standardized treatment across the country, including human C1 esterase inhibitor replacement therapy (Cinryze) and monoclonal antibody therapy (lanadelumab). This centralized and fully funded program has significantly enhanced the detection, registration, and clinical management of HAE, contributing to the elevated proportion of complement deficiencies observed in our study. These findings underscore the influence of targeted national healthcare policies and diagnostic capabilities on the distribution of IEI subtypes and highlight the importance of considering such factors when interpreting epidemiological data.

Severe infections ranged from recurrent bacterial pneumonia and chronic otitis media to persistent fungal skin infections, consistent with previous reports. One notable case involved a child with SCID who experienced recurrent fungal infections of the oral mucosa and skin, aligning with findings documented elsewhere [32]. Another case involved a patient with CGD who suffered multiple abscesses due to *Staphylococcus aureus* [33].

Hospitalizations occurred in 13.3% of patients due to severe infections such as sepsis, osteomyelitis, or meningitis. Malignancies, particularly lymphomas and leukemias, were the second most common cause of death after infections. In HAE, asphyxiation from mucosal swelling was a frequent cause of mortality [34,35,36]. Vaccination-related complications were identified in 25 patients, with disseminated BCG infections and vaccine-associated paralytic poliomyelitis being the most common.

The diverse clinical manifestations of IEIs pose significant diagnostic challenges for GPs, often leading to a “diagnostic odyssey” due to the rarity and complexity of these conditions. GPs face difficulties in identifying IEIs, as their symptoms can mimic more common diseases, and variability in clinical presentation further complicates diagnosis [32,33,34,35]. Misdiagnosis and delayed care are common due to a lack of training and awareness. To address these challenges, robust patient registration systems are crucial, enabling better tracking of symptoms and facilitating genetic testing, which improves diagnostic accuracy [36]. Advances in genetic testing, like next-generation sequencing, are vital for identifying IEIs, though their complexity requires collaboration between GPs and specialists. A multidisciplinary approach, supported by registries and genetic testing, can significantly enhance patient management and outcomes.

Delays in diagnosis can result in significant complications, as shown by global studies. For example, research carried out in New Zealand involving patients with CVID revealed that prolonged diagnostic delays were notably linked to an increased risk of bronchiectasis development, emphasizing the vital necessity of early identification and prompt referral for immunological evaluation [53]. This discovery endorses the incorporation of diagnostic delay as a regular measure, characterized as the duration from the start of symptoms to a verified diagnosis. Future studies of the national registry in Kazakhstan ought to include this measure to more effectively pinpoint obstacles to healthcare access and comprehend their influence on clinical results.

This research presents several significant limitations that need to be recognized. Initially, while clinical and laboratory criteria were utilized to recognize humoral and other IEI subtypes, established diagnostic guidelines from organizations like ESID or ICON were not uniformly followed. Consequently, there might be differences in classification and diagnostic accuracy, especially for CVID and SIgAD, as overlapping phenotypes and polygenic factors hinder categorization [32,33]. Secondly, the documented incidence of SCID is probably understated due to the absence of national prenatal or newborn screening initiatives. Certain patients may have died before receiving a definitive diagnosis, thus influencing both prevalence and mortality estimates [28,29]. Mortality data related to conditions such as ataxia–telangiectasia and DiGeorge syndrome should also be interpreted with caution, given the young average age of the affected population and the lack of long-term outcome tracking. Moreover, the majority of patients included in this study were children. This pediatric predominance may reflect diagnostic and referral biases, as specialized immunology services are primarily located in pediatric facilities. Consequently, adult patients with milder or late-onset phenotypes, such as CVID or complement deficiencies, may be underdiagnosed or entirely missed. This imbalance highlights a critical gap in adult immunology services and follow-up care, necessitating targeted efforts to strengthen adult IEI detection and surveillance. Diagnostic delays further limit interpretation, particularly for adult-onset disorders like CVID and complement deficiencies, where specialized immunology care remains insufficient. Additionally, limitations in retrospective data made it difficult in many cases to distinguish between symptomatic and incidental diagnoses of SIgAD.

Importantly, while Kazakhstan hosts two key medical institutions involved in the diagnosis and management of IEI—namely, the RDC and the National Center for Maternal and Child Health in Astana, and the Scientific Center of Pediatrics and Pediatric Surgery in Almaty—only the RDC in Astana functions as a specialized immunology center. No such immunology centers currently exist in other regions of the country. This centralization leads to significant geographic disparities in access to care, likely contributing to referral bias and underrepresentation of patients from rural and underserved areas. Ultimately, despite the establishment of a national registry, incomplete regional reporting, limited availability of genetic diagnostics, and variability in data quality reduce the generalizability of the study’s findings.

Furthermore, the interpretation of genetic findings—specifically regarding zygosity and patterns of inheritance—represents an additional constraint. The majority of variants were observed in a heterozygous state; however, not all conform to traditional autosomal dominant inheritance paradigms. The categorization of numerous variants as pathogenic remains ambiguous owing to the lack of segregation analysis or functional confirmation. This issue is particularly pertinent for infrequent variants such as *PI3KCG*, for which existing evidence does not definitively substantiate pathogenicity. These constraints have been meticulously recognized, and interpretations have been rendered with caution, consistent with the ACMG variant classification protocols.

Future plans for addressing the identified challenges in IEI care in Kazakhstan focus on improving diagnostic capacity, expanding access to modern therapies, and enhancing healthcare infrastructure. One key priority is to strengthen genetic diagnosis by incorporating advanced genomic technologies, such as whole-exome sequencing (WES) and next-generation sequencing (NGS), in order to facilitate early and precise identification of IEIs. Establishing partnerships with international genetic research centers and building local capacity among healthcare professionals are also essential. Additionally, there are ongoing efforts to expand access to hematopoietic stem cell transplantation (HSCT) by increasing the number of transplant centers and improving local infrastructure. This includes the development of a national HSCT network to ensure timely referrals and reduce the necessity for patients to seek treatment abroad. Among the survivors with SCID, three received successful HSCT and two were undergoing gene therapy evaluation in other countries. These findings emphasize the critical need for neonatal screening, early diagnosis, and prompt initiation of definitive treatment. Another important future objective is to include SCID screening in the national newborn screening program. Early detection enables timely interventions, such as HSCT, which can greatly improve survival rates and reduce healthcare costs. Achieving these goals will require sufficient funding, targeted training for healthcare providers in genetic and immunological diagnostics, and the establishment of a centralized registry system for data collection and patient monitoring. These combined strategies aim to reduce diagnostic delays, improve access to essential treatments, and enhance the overall quality of life for individuals with IEIs in Kazakhstan.

## 5. Conclusions

This research offers the most exhaustive national examination of IEIs within Kazakhstan to this point, utilizing retrospective registry data amassed over a period of fourteen years. The results emphasize both advancements and ongoing obstacles in the acknowledgment, diagnosis, and treatment of IEIs throughout the nation. Despite Kazakhstan’s significant enhancements in detection rates relative to previous periods, the overall prevalence of 1.36 per 100,000 individuals and an incidence rate of 27.07 per 100,000 live births remain substantially inferior to statistics reported in high-income nations. These inconsistencies may be ascribed to underdiagnosis, restricted access to specialized medical care, and an uneven distribution of diagnostic resources across various regions.

The predominance of pediatric diagnoses, with approximately 72 percent of cases documented in individuals under the age of 18, indicates commendable advancements in the identification of IEIs among younger demographics. Nonetheless, the significantly lower identification rate of adult cases reveals a critical void within the existing healthcare framework. Delays in diagnosis, particularly for conditions such as CVID, SIgAD, and complement deficiencies, point to systemic impediments within adult healthcare environments, which include a deficiency of expertise in clinical immunology and an absence of standardized referral pathways.

The distribution of IEI subtypes in Kazakhstan generally aligns with global patterns, with antibody deficiencies representing the predominant category. However, the ratio of complement deficiencies is markedly elevated compared to data recorded in most international registries. This trend is likely a reflection of the newly instituted government-funded initiative focused on HAE, which has significantly improved both diagnostic accuracy and clinical management through the application of standardized testing and treatment availability. Mortality assessments reveal a disproportionately elevated burden among patients with SCID and syndromic combined immunodeficiencies, where diagnostic delays and restricted access to life-saving interventions such as HSCT are primary contributing factors.

Regional disparities were also notably pronounced, with urban centers such as Astana and Almaty documenting higher incidence rates, while rural and southern areas displayed significantly lower detection rates. These variations are indicative of infrastructural deficiencies, inadequate training for general practitioners in the recognition of IEI symptoms, and a scarcity of molecular diagnostic capabilities outside major urban locales. Although the registry has made considerable progress in data compilation, its coverage continues to be incomplete, particularly in remote regions.

To enhance patient outcomes, Kazakhstan must undertake several strategically targeted reforms. These reforms should encompass the incorporation of SCID screening into the national newborn screening initiative, the expansion of genomic diagnostic platforms such as NGS and WES, the decentralization of immunology services to regional medical facilities, and the establishment of a national HSCT network. Investment in the training of clinicians, patient education, and outreach to underserved populations is crucial to closing diagnostic gaps. The enhancement of the national IEI registry through real-time data reporting and follow-up protocols will improve surveillance and facilitate prompt clinical decision-making.

By prioritizing these initiatives, Kazakhstan has the potential to diminish diagnostic delays, enhance access to medical care, and align its IEI management strategies with international standards, ultimately improving a prognosis and quality of life for individuals suffering from IEIs throughout the nation.

## Figures and Tables

**Table 1:** Registry-based frequency of IEIs in Kazakhstan by gender and age group (data as of 2023).

Parameters	Number of Cases, N	Population	Registry-Based Frequency (per 100,000)
Male	139	9,647,701	1.44
Female	130	10,119,106	1.28
Age at diagnosis			
0–4	107	2,058,952	5.19
5–9	69	1,953,581	3.53
10–14	13	1,822,919	0.71
15–17	6	1,173,320	0.51
Over 18	74	12,758,035	0.58
Total (in register)	269	19,766,807	1.36

Here, the table presents the registry-based frequency of IEIs in Kazakhstan as of 2023, stratified by gender (male and female) and age groups at diagnosis (0–4 years, 5–9 years, 10–14 years, 15–18 years, and over 18 years). The frequency per 100,000 population is calculated based on the number of diagnosed cases and the total population for each category. The overall frequency rate is derived from the cumulative number of registered cases across all groups.

**Table 2:** Distribution of patients by categories and subtypes of IEIs in Kazakhstan.

Types of IEIs	Number of Patients, N	Percentage of Total IEI Cases, %	Male/Female (Ratio)	Genetically Confirmed	Mortality, %
Immunodeficiencies affecting cellular and humoral immunity (*n* = 11)					
SCID	10	3,72%	9:1	9	50%
MHC Class II deficiency	1	0.37%	All male	-	0%
Combined immunodeficiencies with associated or syndromic features (*n* = 36)					
Hyper-IgE syndrome	11	4.09%	10:1	9	9%
WAS	7	2.60%	All male	7	0%
Ataxia–telangiectasia	9	3.35%	8:1	7	22%
DiGeorge syndrome	8	2.97%	7:1	8	25%
Nijmegen breakage syndrome	1	0.37%	All female	-	0%
Predominantly antibody deficiencies (*n* = 120)					
CVID	45	16.73%	26:19	34	2%
SIgAD	43	15.99%	9:34	22	0%
Bruton’s agammaglobulinemia	10	3.72%	All male	3	20%
Functional B-cell deficiency *	22	8.18%	16:6	7	0%
Immune dysregulation diseases (*n* = 3)					
APECED	1	0.37%	All female	1	0%
ALPS	2	0.74%	1:1	1	0%
Congenital defects of phagocyte number or function (*n* = 12)					
CGD	12	4.46%	All male	12	8%
Autoinflammatory disorders (*n* = 1)					
FCAS	1	0.37%	All female	1	0%
Complement deficiencies (*n* = 83)					
C1 inhibitor deficiency	83	30.86%	12:71	66	2%
Defects in intrinsic and innate immunity (*n* = 3)					
TLR-3 deficiency	2	0.74%	1:1	-	0%
Chronic mucocutaneous candidiasis	1	0.37%	All male	-	0%

* Defined as patients with reduced memory B-cell numbers and/or poor vaccine responses without genetic confirmation, in line with ESID functional classification.

**Table 3:** Distribution of IEI among children (0–17 years at diagnosis) by category and subtype in Kazakhstan.

Types of IEIs	Number of Patients, N	Percentage of Total IEI Cases Among Children, %	Male/Female (Ratio)	Genetically Confirmed	Mortality, %
Immunodeficiencies affecting cellular and humoral immunity (*n* = 11)					
SCID	10	5.12%	9:1	9	50%
MHC Class II deficiency	1	0.51%	All male	-	0%
Combined immunodeficiencies with associated or syndromic features (*n* = 36)					
Hyper-IgE syndrome	11	5.64%	10:1	9	9%
WAS	7	3.59%	All male	7	0%
Ataxia–telangiectasia	9	3.34%	8:1	7	22%
DiGeorge syndrome	8	4.10%	7:1	8	25%
Nijmegen breakage syndrome	1	0.51%	All female	-	0%
Predominantly antibody deficiencies (*n* = 58)					
CVID	24	12.31%	15:9	20	4%
SIgAD	24	12.31%	5:19	22	0%
Bruton’s agammaglobulinemia	10	5.13%	All male	3	20%
Functional B-cell deficiency	22	11.28%	16:6	7	0%
Immune dysregulation diseases (*n* = 3)					
APECED	1	0.51%	All female	1	0%
ALPS	2	1.03%	1:1	1	0%
Congenital defects of phagocyte number or function (*n* = 12)					
CGD	12	6.15%	All male	12	8.30%
Autoinflammatory disorders (*n* = 1)					
FCAS	1	0.51%	All female	1	0%
Complement deficiencies (*n* = 49)					
C1 inhibitor deficiency	49	25.13%	6:43	41	2%
Defects in intrinsic and innate immunity (*n* = 3)					
TLR-3 deficiency	2	1.03%	1:1	-	0%
Chronic mucocutaneous candidiasis	1	0.51%	All male	-	0%

**Table 4:** Distribution of IEI among adults (>18 years at diagnosis) by category and subtype in Kazakhstan.

Types of IEIs	Number of Patients, N	Percentage of Total IEI Cases Among Adults, %	Male/Female (Ratio)	Genetically Confirmed	Mortality, %
Predominantly antibody deficiencies (*n* = 40)					
CVID	21	28.38%	11:10	14	0%
SIgAD	19	25.68%	4:15	0	0%
Complement deficiencies (*n* = 34)					
C1 inhibitor deficiency	34	45.95%	6:28	25	3%

**Table 5:** Genetic variants identified in Kazakhstani patients with IEIs.

Clinical Diagnosis	Gene	Variant (HGVS)	Zygosity	ACMG Classification	Inheritance	Genetically Tested Cases/Total (%)
SCID	*IL2RG*, *JAK3*, *IL7R*, *PTPRC*, *RAG1*, *AK2*, *CD40LG*	c.678delA; p.Glu226Lysfs*5	Hemizygous	Pathogenic	X-linked	9/10 (90%)
Combined immunodeficiencies with syndromic features	*DOCK8*	c.2916_2919del; p.Glu972fs	Heterozygous	Pathogenic	Autosomal recessive	9/9 (100%)
Hyper-IgE syndrome	*STAT3*	c.1144C>T; p.Arg382Trp	Heterozygous	Likely pathogenic	Autosomal dominant	9/11 (81.8%)
WAS	*WAS*	c.271C>T; p.Arg91 *	Heterozygous	Pathogenic	X-linked	7/7 (100%)
Ataxia–telangiectasia	*ATM*	c.5932G>A; p.Glu1978Lys	Heterozygous	Pathogenic	Autosomal recessive	7/9 (77.8%)
DiGeorge syndrome	*TBX1*	22q11 deletion	Heterozygous	Pathogenic	Microdeletion	8/8 (100%)
Nijmegen breakage syndrome	*NBN*	c.657del5	Heterozygous	Pathogenic	Autosomal recessive	1/1 (100%)
CVID	*TNFRSF13B*, *CR2*, *PIK3R1*, *TRNT1*, *IRF2BP2*, *RAC2*, *PIK3CG*, *NFKB2*	c.310T>C; p.Cys104Arg	Heterozygous	VUS (*n* = 33), Pathogenic (*n* = 1), Likely Pathogenic (*n* = 0)	Mostly Autosomal dominant *	34/45 (75.6%) †
SIgAD	Unknown	Not identified	Unknown	VUS	Unknown	22/43 (51.2%)
Bruton’s agammaglobulinemia	*BTK*	c.1483G>A; p.Glu495Lys	Heterozygous		X-linked	3/10 (30%)
Functional B-cell deficiency	*BLNK*	c.736G>A; p.Glu246Lys	Heterozygous	Pathogenic	Autosomal dominant	7/22 (31.8%)
APECED	*AIRE*	c.769C>T; p.Arg257 *	Heterozygous	Pathogenic	Autosomal dominant	1/1 (100%)
ALPS	*FAS*	c.841T>C; p.Phe281Leu	Heterozygous	Pathogenic	Autosomal dominant	1/2 (50%)
Phagocytic cell defects	*CYBB*	c.469C>T; p.Arg157Cys	Heterozygous	Pathogenic	X-linked	12/12 (100%)
FCAS	*NLRP3*	c.1009A>G; p.Thr337Ala	Heterozygous	Pathogenic	Autosomal dominant	1/1 (100%)
HAE	*SERPING1*	c.1396C>T; p.Arg466Cys	Heterozygous	Pathogenic	Autosomal dominant	66/83 (79.5%)
TLR-3 deficiency	*TLR3*	c.1660C>T; p.Arg554 *	Heterozygous	Pathogenic	Autosomal recessive	1/2 (50%)
Chronic mucocutaneous candidiasis	*STAT1*	c.800C>T; p.Thr267Ile	Heterozygous	Pathogenic	Autosomal dominant	1/1 (100%)

“*” Although variants were identified in heterozygous form, inheritance patterns remain inconclusive for several CVID-associated genes. “†” Only one CVID patient had a confirmed pathogenic variant; all others harbored VUS.

**Table 6:** Vaccine-related complications in IEI patients.

Number of Patients, N	Clinical Diagnosis	Vaccine-Related Complication	Clinical Outcome
2	SCID	Disseminated BCG infection with lymphadenitis	Recovered with prolonged antimycobacterial therapy
3	SCID	Disseminated BCG with osteomyelitis	Ongoing follow-up; improved on treatment
1	SCID	Disseminated BCG with skin and lung involvement	Survived with mild residual lung damage
1	SCID	Paralytic poliomyelitis post-OPV	Stable with residual lower limb weakness
1	MHC Class II deficiency	Disseminated BCG with persistent fever	Managed successfully; immune workup ongoing
2	Hyper-IgE syndrome	Local BCG reactivation with cold abscess	Resolved with antibiotic therapy
4	WAS	BCG lymphadenitis with systemic symptoms	Improved with supportive care
2	Ataxia–telangiectasia	Disseminated BCG with pneumonia	Survived with chronic bronchial changes
2	DiGeorge syndrome	BCG osteomyelitis of the spine	Stable with physical therapy
1	Nijmegen breakage syndrome	BCG reactivation with skin granulomas	Controlled with long-term antimycobacterial therapy
3	CVID	BCG lymphadenitis with delayed healing	Recurrent local symptoms; stable
3	Bruton’s agammaglobulinemia	Vaccine-associated paralytic poliomyelitis	Residual motor deficit

## Data Availability

The original contributions presented in this study are included in the article/Supplementary Material. Further inquiries can be directed to the corresponding author.

## Supplementary Materials

The following supporting information can be downloaded at: https://www.mdpi.com/article/10.3390/jcm14155353/s1, Supplementary File S1: Demographics and Healthcare System of Kazakhstan.
