# Supplementary material for: Registry-Based Frequency and Clinical Characteristics of Inborn Errors of Immunity in Kazakhstan: A Retrospective Observational Cohort Study (2009–2023)
Source: J Clin Med. 2025 Jul 29;14(15):5353. doi: 10.3390/jcm14155353 (PMC12347398; doi:10.3390/jcm14155353)
Supplement: Supplementary file 1 [file jcm-14-05353-s001.zip › jcm-3666262-supplementary.pdf]

2.1. Demographics and healthcare system of Kazakhstan

Kazakhstan is a country in Central Asia and Eastern Europe with a population of 19,944,726 people in 2023, divided into 17 regions [22]. As of 2023, Kazakhstan has a diverse population, consisting of individuals of Kazakh (70.4%), Russian (15.5%), Uzbek (3.2%), Ukrainian (2.0%), Uighur (1.5%), and German (2%) origins, along with other ethnic groups (5%) [23]. The country has established a three-tier healthcare system, comprising primary, secondary, and tertiary levels of medical care.

Kazakhstan has established a comprehensive three-level healthcare system designed to provide efficient and accessible medical care to its population. This system ensures that patients receive appropriate care based on the complexity of their medical needs, with a clear referral pathway from primary to tertiary care. Below is an enhanced and more structured description of each level of care, along with additional relevant information [24-26]:

Primary level of care. The primary level focuses on delivering essential healthcare services, acting as the first point of contact for patients. Primary health care (PHC) specialists, including general practitioners (GPs), therapists, pediatricians, and family doctors, provide medical care in outpatient settings, hospital-replacing conditions, and even at patients' homes when necessary. The key responsibilities of PHC include preventive care, early disease detection, chronic disease management, and health education. This level of care is crucial for reducing the burden on specialized and hospital-based services by addressing a wide range of common health issues early and efficiently.

Secondary level of care. The secondary level provides specialized medical care for more complex or specific health conditions that cannot be managed at the primary level. Patients are typically referred to this level by PHC specialists. Specialized care is delivered by doctors trained in specific areas such as cardiology, endocrinology, surgery, and obstetrics. Services at this level are available in both outpatient and hospital-based settings, allowing for more targeted interventions that require specialized expertise, diagnostic tools, or treatments.

Tertiary level of care. Tertiary care is the highest and most advanced level of medical care in Kazakhstan's healthcare system. It focuses on providing highly specialized services that often involve high-tech medical equipment and advanced treatment protocols. This level of care is typically accessed through referrals from the primary or secondary levels and is delivered in specialized hospitals and research centers. Tertiary care is essential for managing severe or rare diseases, performing complex surgeries, and offering cutting-edge treatments such as organ transplants and oncology therapies.

The healthcare system is funded through a hybrid model that includes government budget allocations and a compulsory social health insurance system (MSHI), introduced in 2020. According to this model, the government ensures a fundamental set of complimentary medical services for all citizens and legal residents, encompassing emergency care, primary health care, and disease prevention services. Extra services, including expert care, diagnostic tests, and hospital stays, are included under the MSHI for those who are insured. Uninsured patients might have to cover costs themselves or rely on

restricted state-sponsored services. Private healthcare options are available as well, but are generally paid directly by the patient or through insurance provided by employers.

Kazakhstan’s three-level healthcare system is designed with a referral mechanism in place, ensuring that patients are directed to the appropriate level of care based on their health needs. PHC specialists act as gatekeepers, managing common health conditions and referring patients to specialized services when necessary. The secondary level often manages more serious or chronic conditions, while the tertiary level handles the most complex and high-risk cases.
